# The effect of first‐person perspective action observation training on upper extremity function and activity of daily living of chronic stroke patients

**DOI:** 10.1002/brb3.2565

**Published:** 2022-04-10

**Authors:** Ji‐Ae Yu, JuHyung Park

**Affiliations:** ^1^ Department of Occupational Therapy Cheongju Mary's Hospital Cheongju Korea; ^2^ Department of Occupational Therapy College of Health and Medical Sciences Cheongju University Cheongju Korea

**Keywords:** ADL, motor function, stroke

## Abstract

The purpose of this study was to investigate the effects of First‐Person Perspective Action Observation training and Third‐Person Perspective Action Observation training on upper extremity function and activities of daily living of patients with stroke‐induced hemiplegia.

This was a single‐blind randomized study of 20 stroke patients (more than 6 months after the incident stroke) with upper extremity disabilities. The subjects who satisfied the inclusion and exclusion criteria were randomly divided into two groups: First‐Person Perspective Action Observation training group and Third‐Person Perspective Action Observation training group. The measurements were performed using Action Research Arm Test (ARAT) and Korean Modified Barthel Index (K‐MBI) and Motor Activity Log (MAL).

The results of this study showed statistically significant differences (*p *< .05) in the upper extremity function and activity of daily living after the intervention in all two groups. Upon comparison of the amount of change between the experimental group and the control group, there was significant difference in upper extremity function and activity of daily living (*p *< .05).

Action Observation training was found to have an effect on the upper extremity function and activity of daily living on chronic stroke patients. First‐Person Perspective Action Observation training was more effective in improving upper limb function and activity of daily living than the Third‐Person Perspective Action Observation training.

## Abstract 2

This study is a clinical trial study to investigate the effects of first‐person action observation training and third‐person action observation training on upper extremity function and daily living performance of stroke patients.

## 1 INTRODUCTION

Stroke patients experience complex forms of impairments of motor functions and cognitive functions, usually affecting one half of the body, including paralysis, muscle weakness, speech impairment, sensory impairment, and cognitive impairment, caused by the infarction or hemorrhage of cerebral blood vessels due to circulatory disorders affecting the cerebral blood supply (Zwecker et al., 2002). This physical damage and disability commonly appear in the form of hemiplegia (Page et al., 2001). Approximately 85% of stroke patients experience hemiplegia, ≥65% of whom will experience upper extremity dysfunction (Wolf et al., 2001). Only about 5% of these patients show complete recovery of upper extremity function, and 20% recover partially (Hayward et al., 2010).

Upper extremity function plays a significant role in daily activities requiring delicate movements (e.g., feeding, washing, dressing, and writing), ambulation, balancing, and protective reflexes (Invernizzi et al., 2013). Thus, in the presence of upper extremity function impairment, the activities of daily life can become more complicated, and the quality of life can decrease (Kamper et al., 2002).

In the chronic stage after a stroke, the recovery of upper extremity function is slower than the recovery of lower extremity function (Hayward et al., 2010) and tends to be partial because the location or the degree of damages may differ. In addition, daily activities can often be performed using only the nonparalyzed side of the upper extremities, whereas functional motion is only possible using both lower extremities (Taub et al., 1993). Thus, less daily use of the paralyzed upper extremity than the paralyzed lower extremity tends to reduce the stimulations to the nerve pathways in the brain responsible for the upper extremities, slowing the promotion of neuroplasticity (Feydy et al., 2002).

Previous studies have investigated interventions, including traditional rehabilitation therapy, constraint‐induced movement therapy (CIMT), upper extremity function training using robots, virtual reality training, and electromyography‐triggered neuromuscular electrical stimulation, to improve the upper extremity functions in chronic stroke patients (Oujamaa et al., 2009). Additional interventions based on the mirror neuron theory, such as mirror therapy, imagination training, and action observation training (AOT), are also being tested if they assist the recovery of upper extremity functions and improve the activity of daily life performance in patients with stroke (Eaves et al., 2016).

A mirror neuron is an area of the brain activated when performing purposeful movements and while observing movements performed by other entities, and it was first discovered in the F5 region corresponding to the ventral premotor cortex of a monkey (Gallese et al., 1996). The human mirror neuron is involved in the perception and comprehension of the actions performed by others (Buccino et al., 2001). It activates the frontal–parietal lobe network, a mimic circuit of the brain, through visual projection and is also an essential part of the brain that works when trying to imitate actions observed visually in an attempt to learn them (Rizzolatti et al., 1998). The term signifies “reflecting like a mirror” by observing other people's actions, in which the observer will feel as if acting on their own (Maeda et al., 2002). They are activated more when observing hand movements, such as gripping, tearing, grabbing, and manipulating (Rizzolatti et al., 1998).

In mirror neuron theory, the brain activity and the response appear differently depending on the observation perspective (Watanabe et al., 2011). According to a study by Pelosin et al. (2013), which investigated cerebral activity response according to the observation perspective using brain imaging techniques, the brain activity response similar to when moving one's limbs appeared in first‐person perspective action observation (Pelosin et al., 2013). In contrast, the brain activity response similar to when observing other people's actions appeared during third‐person perspective action observation. Moreover, the observer's limbs anatomically matched the model's limbs in the video in first‐person perspective action observation, while the model's limbs in the video appeared as a mirror image in third‐person perspective action observation(Pelosin et al., 2013). Jackson et al. (2006), on the basis of observing the behaviors of healthy participants, compared the brain activity mechanisms while mimicking the observed movements. The authors reported that first‐person observation showed increased activation in the sensorimotor cortex relative to third‐person observation. Similarly (Jackson et al., 2006), Watanabe et al. (2011) compared differences in the brain activity response depending on the observation perspective of the healthy participants by brain imaging techniques. Localized and selective cerebral activation was higher during first‐person perspective action observation than third‐person perspective action observation (Watanabe et al., 2011).

However, many studies lack first‐person perspective action observation of stroke patients despite its advantages over third‐person perspective action observation. In addition, from a therapy standpoint, no study compared first‐person perspective action observation training with the third‐person perspective regarding their effects on upper extremity functions and activities of daily living of stroke patients. Here, this study investigated the effects of first‐person and third‐person perspective observation training on upper extremity function and activities of daily living in patients with chronic stroke.

## 2 METHODS

### 2.1 Participants

Among the stroke patients treated at the S Hospital in Cheongju‐si, Chungcheongbuk‐do from June 2020 to September 2020, we recruited 20 participants who met the criteria for selection. All participants consented to participate and understood the purpose of this study. We randomly assigned 10 participants to the experimental group who received first‐person perspective action observation training and the other 10 to the control group who received third‐person perspective action observation training. All participants followed the procedure approved by the Institutional Review Board of Cheongju University (1041107‐202004‐HR‐013‐01). Participants of this study were recruited by the following selection and exclusion criteria.

The selection criteria for this study were (1) patients with stroke diagnosed by magnetic resonance imaging (MRI) or computed tomography (CT) evaluation over 6 months ago. (2) Understanding the instructions provided by the researcher and the ability to follow them with Korean Mini‐Mental Exam (K‐MMSE) score ≥ 24. (3) The Brunnstrom Recovery Stage (BRS) 3 or higher with increased stiffness and voluntary joint movements in the paralyzed hand and the arm and being able to perform the intervention tasks, such as hook grasp and mass grasp. (4) No abnormal visual perception, including hemispatial neglect, as demonstrated by the Motor‐free Visual Perception Test (MVPT) result. (5) Those who understood the purpose of this study and consented to participate. Exclusion criteria were those with (1) mental or neurological diseases, (2) pain in the paralyzed upper extremity and impaired proprioception, and (3) participation in another study.

### 2.2 Procedures

This study used a two‐group randomized pre‐/postexperimental design with a computer program used for randomization. The study proceeded without information about which group between the experimental group and the control group the subjects of the study belong to. All subjects participated equally in the traditional occupational therapy and exercise therapy programs along with the interventions of this study. Three occupational therapists with > 2 years of clinical experience assisted in the pre‐evaluation, intervention, and postevaluation. All subjects were provided with an intervention program of 20 interventions, 30 minutes daily, 5 times a week, for 4 weeks. The gender, age, height, weight, date of stroke onset, location of the lesion, and type of paralysis were looked up in medical records. All subjects were evaluated by the K‐MMSE, BRS, and MVPT.

After 20 subjects who met the screening criteria were selected, they were divided randomly into the experimental group (*n* = 10) receiving first‐person perspective action observation training and the control group (*n* = 10) receiving third‐person perspective action observation training. Before the intervention, their upper extremity function was evaluated by ARAT, and K‐MBI and MAL were used to evaluate their activities‐of‐daily‐living performance to confirm the homogeneity between the two groups. These assessments were repeated after the intervention.

In the experimental group, first‐person perspective action observation training was provided separately after the conventional occupational therapy. Occupational therapy was performed for 30 minutes daily, 5 times a week, for 4 weeks. First‐person perspective action observation training was conducted for 30 minutes daily, 5 times a week, for 4 weeks. After the conventional occupational therapy, third‐person perspective action observation training was provided separately in the control group. Occupational therapy was provided for 30 minutes daily, 5 times a week, for 4 weeks, and third‐person perspective action observation training was provided for 30 minutes daily, 5 times a week, for 4 weeks. For both groups, the recording of each task was edited to be 5 minutes in length, so the subject observed the video for 5 minutes and then tried to imitate the task for 10 minutes.

**FIGURE 1:**
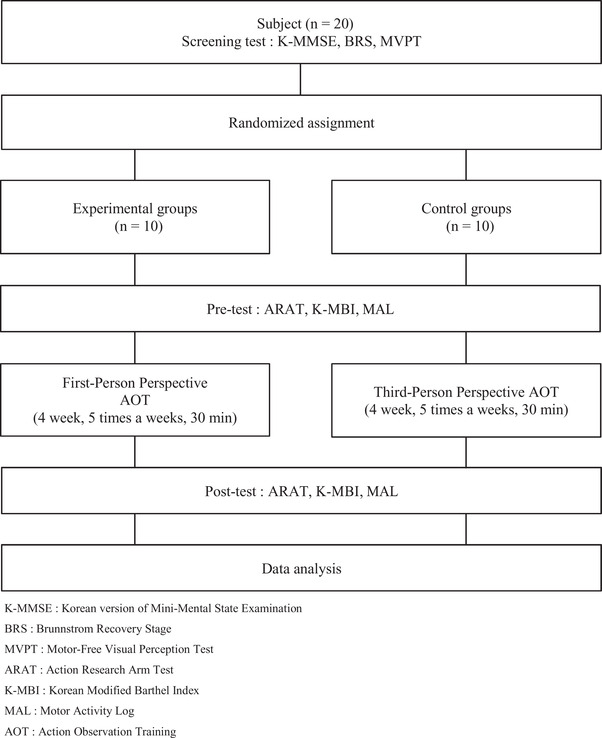
Flow chart of the study

Three occupational therapists administered the conventional occupational therapy interventions that were applied to both study groups at S Hospital in Cheongju‐si, Chungcheongbuk‐do, including the primary author of this article, who assessed the activities of daily living and administered the upper extremity function tests before and after the treatment to all patients to maintain consistency. The research design of this study is shown in Figure 1.

### 2.3 Evaluation tools

The evaluation tools used for selecting study participants included the Korean version of the Mini‐Mental State Examination (K‐MMSE), which was used to assess the cognitive ability related to understanding our interventions and following instructions. The Brunnstrom Recovery Stage (BRS) and Motor‐free Visual Perception Test (MVPT) evaluated the paralyzed upper extremity functions and visual perception ability, respectively. The Action Research Arm Test (ARAT) was used to assess the upper extremity function before and after the intervention, and performance of activities of daily living was evaluated by the Korean Modified Barthel Index (K‐MBI) and the Motor Activity Log (MAL).

The ARAT is an evaluation tool used to measure the upper extremity function of stroke patients with a maximum score of 57; the higher the score, the higher the performance level. There are a total of 19 evaluation items in four categories: grasp (18 points), grip (12 points), pinch (18 points), and action (gross movement: 9 points). Each item consists of a 4‐point scale: 0 for inability to perform the test, 1 for partial performance, 2 for performance with a delay or the presence of struggles in performance, and 3 for average performance. Lang et al. (2006) reported that the test–retest reliability and the intertester reliability of the ARAT were 0.99 and 0.98, respectively, for stroke patients(Lang et al., 2006).

K‐MBI is an instrument used to evaluate the ability to perform activities of daily living, which was standardized after modifying the 5th version of the MBI (Shah et al., 1989) to make it more suitable for use in Korea. The evaluation consists of 10 items: personal hygiene (grooming), feeding, bathing, toilet transfer, stair climbing, dressing, bowel control, bladder control, ambulation, and chair/bed transfers. Each item consists of a 5‐point scale ranging from 0 to the maximum 100; the higher the score, the higher the performance level. Its test–retest reliability and the intertester reliability have been reported to be 0.89 and 0.95, respectively, which are relatively high (Young et al., 2007).

The MAL is a measurement method based on semistructured interviews and is used to learn about the use of the paralyzed arm. It consists of 30 activities of daily living for an examiner to determine the level of the patient's independent use of the paralyzed upper extremity (Page et al., 2001; van der Lee et al., 2004). Each item is a complex task involved in performing the basic and instrumental activities of daily life that measures the Amount of Use (AOU) and the Quality of Movement (QOM) of using the paralyzed arm for each activity. The AOU scoring scale ranges from 0 (“did not use the paralyzed arm at all”) to 5 (“used the paralyzed arm as much as before the stroke”). The QOM scoring scales ranged from 0 (“cannot use the paralyzed arm for activity”) to 5 (“can use the paralyzed arm like before the stroke”). All items were scored on a 6‐point scale; the average score (0−5 points) for all 30 activities was calculated. For the internal consistency, Cronbach's alpha was α = 0.81–0.87 and the test‐retest reliability was 0.91 (Uswatte et al., 2005).

### 2.4 Intervention

The video used in this study consisted of items easily applicable to daily lives. It was selected from upper extremity activity tasks. Both the experimental and control groups observed the video. The 10 tasks were as follows: folding a towel, using scissors, opening and closing an airtight square container, drinking water, making a phone call, squeezing toothpaste on a toothbrush, using a lever spray, using a lever faucet, plugging a cord into an outlet, and opening a bottle cap. Participants sat in a chair in an isolated room without external disturbances and observed the video on a 24‐inch computer screen, 30 cm in front of their position.

**FIGURE 2:**
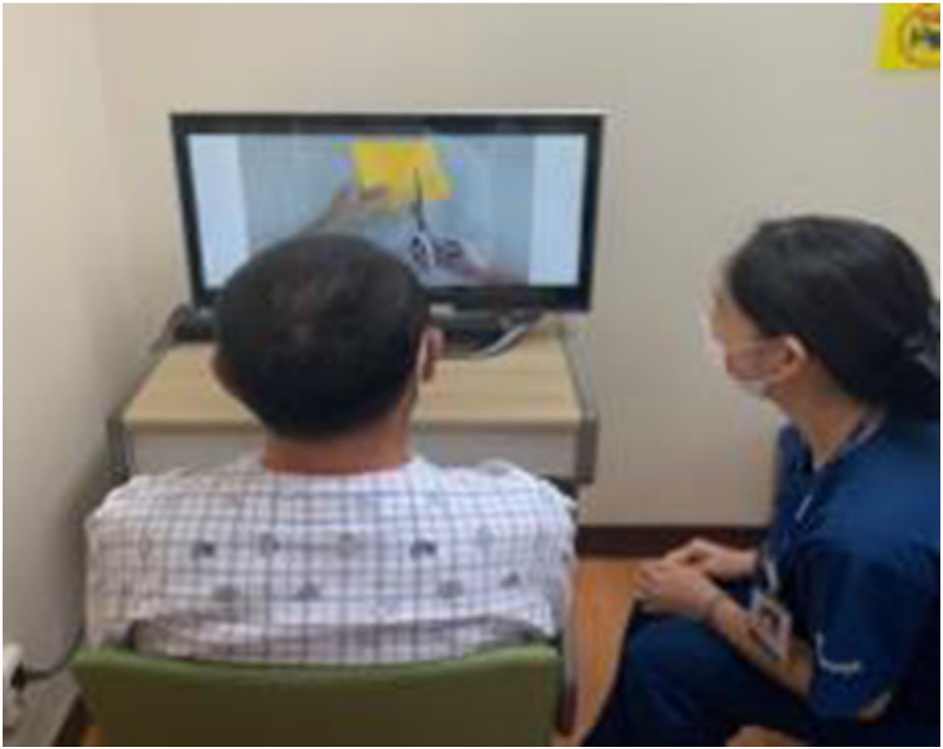
Observing the video

The therapist provided only a brief verbal explanation about the characteristics of tasks and actions to promote observer's concentration while watching the video. Each viewer was asked to imitate the observed action using the object they saw in the video (Figure 2).

**FIGURE 3:**
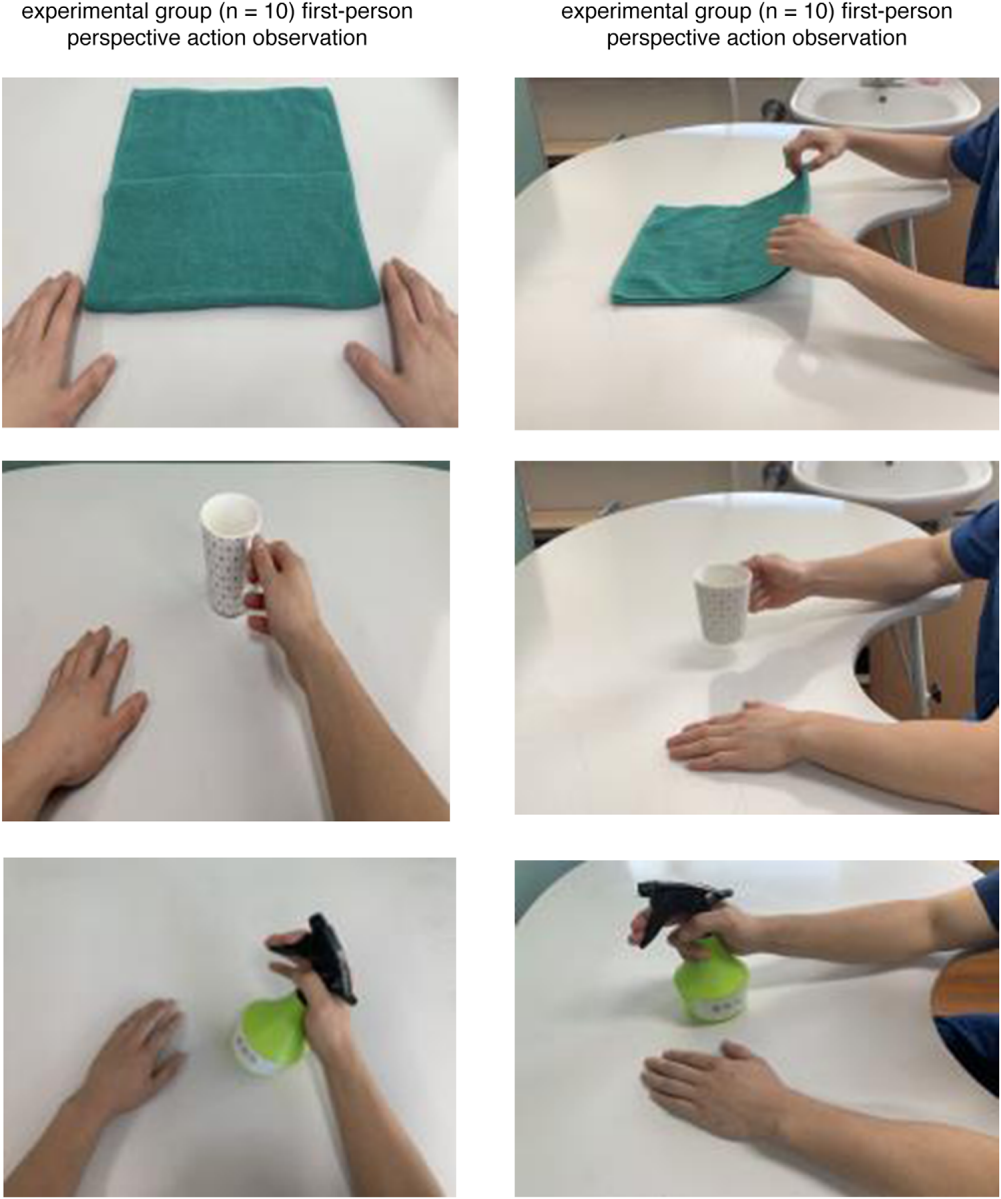
Action observation training program

Tasks of action observation training that the experimental group undertook were recorded in the first‐person perspective. Tasks in action observation training that the control group undertook were recorded from a third‐person perspective from front to side (Figure 3).

**FIGURE 4:**
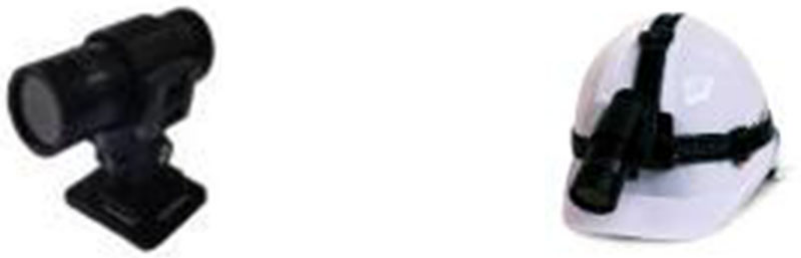
The device for making videos

**FIGURE 5:**
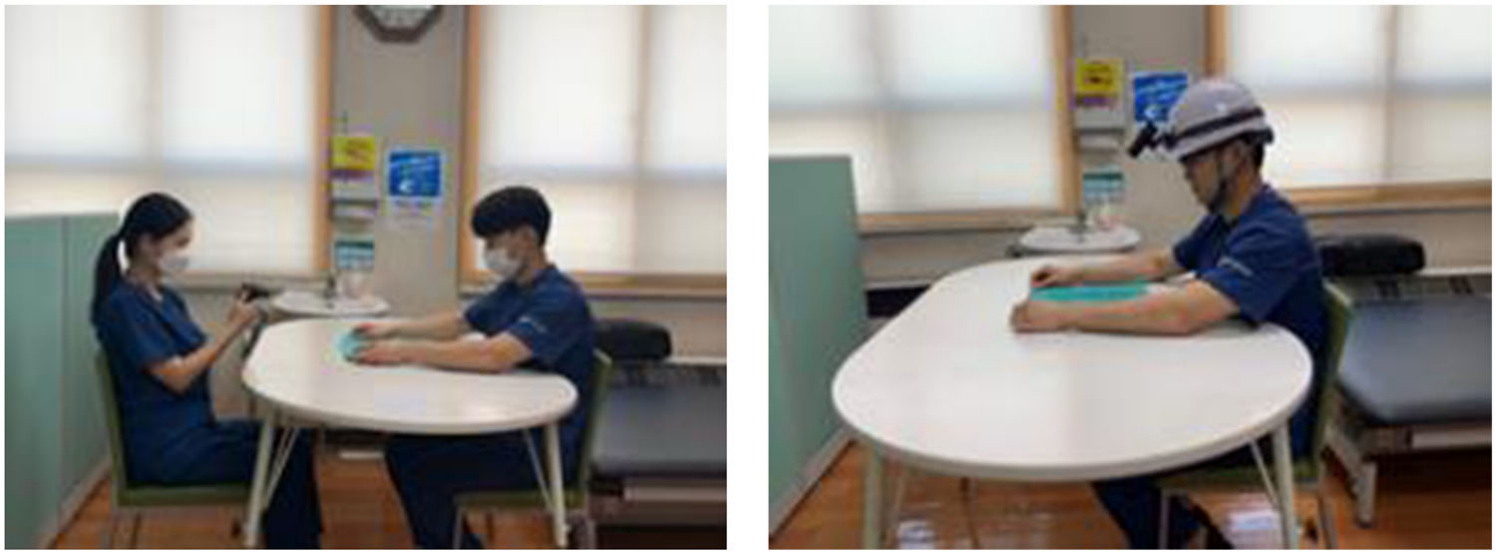
Experimental group and control group imaging method

To record the action observation training video in this study, an action camera (F9, KSD‐MINI, China) was used. The third‐person perspective video was recorded of the continuous movement of the upper extremities from the front and side of a participant performing the task. The action camera was fixed at the observer's eye level for the first‐person perspective video. An industrial helmet with a head mount band was used to fix the action camera at eye level (Figures 4 and 5).

### 2.5 Data analysis

The data collected in this study were analyzed using the SPSS 22.0 Windows version. The Shapiro‐Wilk test was performed to test the normality of the general data of the subjects. Nonparametric analysis was performed because the normality assumption was not satisfied. The chi‐square test, descriptive statistics, and Mann–Whitney U test were used to analyze the general characteristics and the upper extremity function and daily living performance of the two groups before the intervention. The Wilcoxon signed‐rank test compared the before and after between the experimental group and the control group. The Mann–Whitney U test was conducted to compare the difference between groups in the amount of change in scores on upper extremity function and daily living performance after intergroup intervention. The statistical significance was set to α = .05.

## 3 RESULTS

The general characteristics of the participants are shown in Table 1. The general characteristics of the two groups are not significantly different from each other (*p *> .05).

**TABLE 1:** General characteristics of subjects (*N* = 20)

		Experimental group	Control group		
		(*n* = 10)	(*n* = 10)		
		Mean ± SD/(%)	Mean ± SD/(%)	*z*/*x* ^2^	*p*
Sex (%)	Male	6 (60.0%)	7 (70.0%)	0.22	.63
	Female	4 (40.0%)	3 (30.0%)		
Age (year)		62.20 ± 7.64	61.40 ± 9.33	−0.37	.70
Height (cm)		163.30 ± 8.66	165.80 ± 8.61	−0.49	.62
Weight (kg)		63.40 ± 7.84	66.90 ± 9.89	−0.87	.38
Duration (month)		10.30 ± 2.86	11.30 ± 4.08	−0.38	.70
Type	Infarction	6 (60.0%)	6 (60.0%)	0.0	1.00
	hemorrhagic	4 (40.0%)	4 (40.0%)		
Paralysis	Left	5 (50.0%)	6 (60.0%)	0.20	.65
	Right	5 (50.0%)	4 (40.0%)		
K‐MMSE		26.90 ± 2.60	27.60 ± 2.59	−0.62	.52
MVPT		34.30 ± 2.26	35.00 ± 2.30	−0.58	.56
BRS		4.30 ± 0.82	4.20 ± 0.78	−0.32	.74

*Note*: Values are expressed as mean ± SD.

Abbreviations: BRS, Brunnstrom Recovery Stage; K‐MMSE, Korean version of Mini‐Mental State Examination; MVPT, Motor‐Free Visual Perception Test.

* Significant difference (*p *< .05).

** Significant difference (*p *< .01).

The upper extremity functions before the intervention were compared between the two groups. There was no significant difference between the two groups on the overall ARAT score (*p *> .05). Similarly, no significant difference was found between the two groups in terms of the subitems of the ARAT (*p *> .05) (Table 2).

**TABLE 2:** Comparison of upper extremity function scores before the intervention between the groups (*N* = 20)

	Experimental group	Control group		
	(*n* = 10)	(*n* = 10)		
ARAT	Mean ± SD	Mean ± SD	*z*	*p*
Grasp	12.80 ± 3.19	12.10 ± 2.28	−0.64	.51
Grip	9.10 ± 2.51	8.90 ± 2.98	−0.07	.93
Pinch	7.30 ± 3.12	7.40 ± 3.59	−0.19	.84
Gross movement	7.30 ± 1.05	7.80 ± 1.87	−1.54	.12
Total score	36.80 ± 9.80	36.10 ± 9.93	−0.22	.82

*Note*: Values are expressed as mean ± SD.

Abbreviation: ARAT, Action Research Arm Test.

* Significant difference (*p* < .05).

** Significant difference (*p* < .01).

The pre‐/postintervention difference in the ARAT scores was compared between the two groups. A significant difference in the total score was found in both groups (*p < *.05). When comparing subitems of ARAT, the experimental group showed a significant improvement in all items (*p *< .05). The control group showed a significant improvement for grasp, grip, and gross movement (*p *< .05), but not for pinch (*p *> .05) (Table 3).

**TABLE 3:** Action Research Arm Test of the subjects (*N* = 20)

ARAT		Pre Mean ± SD	Post Mean ± SD	*z*	*p*
Experimental group (*n* = 10)	Grasp	12.80 ± 3.19	14.80 ± 2.78	−2.71	.00**
	Grip	9.10 ± 2.51	10.70 ± 2.21	−2.55	.01*
	Pinch	7.30 ± 3.12	8.70 ± 3.46	−2.64	.02*
	Gross Movement	7.30 ± 1.05	8.90 ± 1.13	−2.72	.00**
	Total score	36.80 ± 9.80	41.40 ± 9.53	−2.81	.00**
Control group (*n* = 10)	Grasp	12.10 ± 2.28	12.70 ± 2.16	−2.12	.03*
	Grip	8.90 ± 2.99	9.30 ± 2.86	−2.00	.04*
	Pinch	7.40 ± 3.59	8.00 ± 3.68	−1.89	.05
	Gross Movement	7.80 ± 1.87	8.40 ± 1.35	−2.12	.03*
	Total score	36.10 ± 9.93	37.40 ± 9.73	−2.58	.01*

*Note*: Values are expressed as mean ± SD.

Abbreviation: ARAT, Action Research Arm Test.

* Significant difference (*p *< .05).

** Significant difference (*p *< .01).

Comparison of the postintervention changes in upper extremity function scores between the two groups revealed a significant difference in total ARAT scores between the two groups (*p *< .05). There was a significant difference in the grasp and gross movement out of the subitems (*p *< .05), but not in pinching (*p *> .05) (Table 4).

**TABLE 4:** Comparison of changes in upper extremity function scores after the intervention between the groups (*N* = 20)

	Experimental group	Control group		
	(*n* = 10)	(*n* = 10)		
ARAT	Mean ± SD	Mean ± SD	*z*	*p*
Grasp	2.00 ± 2.05	0.60 ± 0.69	−2.13	.03*
Grip	1.60 ± 1.50	0.40 ± 0.51	−2.26	.02*
Pinch	1.40 ± 2.17	0.60 ± 0.96	−0.95	.34
Gross Movement	1.40 ± 0.69	0.60 ± 0.69	−2.25	.02*
Total score	4.60 ± 4.57	1.30 ± 1.15	−2.74	.00**

*Note*: Values are expressed as mean ± SD.

Abbreviation: ARAT, Action Research Arm Test.

* Significant difference (*p *< .05).

** Significant difference (*p *< .01).

The comparison of the ability to perform activities of daily living between the two groups revealed no preintervention groups difference in the total score on the K‐MBI (*p *> .05). There was no significant difference in all subitems of the K‐MBI (*p *> .05) or the AOU and QOM items of the MAL between the two groups (*p *> .05) (Table 5).

**TABLE 5:** Comparison of activities of daily living scores before the intervention between the groups (*N* = 20)

		Experimental group	Control group		
		Mean ± SD	Mean ± SD	*z*	*p*
K‐MBI	Personal hygiene	2.90 ± 0.73	3.30 ± 0.48	−1.35	.17
	Bathing self	1.90 ± 1.19	2.10 ± 1.19	−0.382	.70
	Feeding	5.90 ± 1.44	5.20 ± 1.68	−1.07	.28
	Toilet	3.50 ± 1.58	4.10 ± 1.44	−0.890	.37
	Stair climbing	1.90 ± 1.96	3.00 ± 1.41	−1.40	.16
	Dressing	3.80 ± 2.09	4.20 ± 1.31	−0.076	.44
	Bowel control	8.80 ± 1.03	8.66 ± 0.96	−0.457	.64
	Bladder control	6.70 ± 1.88	6.30 ± 1.88	−0.418	.67
	Ambulation	8.40 ± 1.26	8.40 ± 1.26	0.000	1.00
	Transfer	9.20 ± 1.93	10.00 ± 2.1	−0.890	.37
	Total score	60.60 ± 12.16	65.10 ± 11.68	−0.60	.54
MAL	AOU	67.60 ± 19.30	66.60 ± 19.87	−0.03	.97
	QOM	71.50 ± 18.73	68.20 ± 19.82	−0.53	.59

*Note*: Values are expressed as mean ± SD.

Abbreviations: AOU, amount of use; K‐MBI, Korean Modified Barthel Index; MAL, Motor Activity Log; QOM, quality of movement.

* Significant difference (*p *< .05).

.** Significant difference (*p *< .01).

The comparison of pre‐/postintervention score differences for the K‐MBI within the groups revealed significant improvements in both the experimental and control groups in terms of the total score of the K‐MBI (*p* < .05). Out of all of the subitems, a significant improvement was found in four subitems (personal hygiene, feeding, toilet transfer, and dressing) in the experimental group (*p* < .05). In contrast, only two subitems, that is, personal hygiene and dressing, showed significant improvements in the control group (*p* < .05) (Table 6).

**TABLE 6:** Korean Modified Barthel Index of the subjects (*N* = 20)

K‐MBI		Pre Mean ± SD	Post Mean ± SD	*z*	*p*
Experimental group (*n* = 10)	Personal hygiene	2.90 ± 0.73	3.60 ± 0.51	−2.33	.02*
	Bathing self	1.90 ± 1.19	2.50 ± 1.08	−1.73	.08
	Feeding	5.90 ± 1.44	7.60 ± 1.50	−2.12	.03*
	Toilet	3.50 ± 1.58	4.70 ± 0.94	−2.00	.04*
	Stair climbing	1.90 ± 1.96	2.50 ± 1.50	−1.73	.80
	Dressing	3.80 ± 2.09	6.80 ± 2.09	−2.42	.01*
	Bowel control	8.80 ± 1.03	9.60 ± 0.84	−1.63	.10
	Bladder control	6.70 ± 1.88	7.20 ± 2.04	−1.34	.18
	Ambulation	8.40 ± 1.26	8.80 ± 1.68	−1.00	.31
	Transfer	9.20 ± 1.93	10.9 ± 1.66	−1.73	.08
	Total score	60.60 ± 12.16	69.70 ± 9.59	−2.81	.00**
Control group (*n* = 10)	Personal hygiene	3.30 ± 0.48	3.80 ± 0.42	−2.23	.02*
	Bathing self	2.10 ± 1.19	2.70 ± 0.94	−1.73	.08
	Feeding	5.20 ± 1.68	6.80 ± 1.54	−1.85	.06
	Toilet	4.10 ± 1.44	5.00 ± 1.41	−1.73	.08
	Stair climbing	3.00 ± 1.41	3.00 ± 1.41	0.00	1.00
	Dressing	4.20 ± 1.31	5.60 ± 1.89	−2.12	.03*
	Bowel control	8.66 ± 0.96	9.20 ± 1.03	−1.34	.18
	Bladder control	6.30 ± 1.88	6.30 ± 1.88	0.00	1.00
	Ambulation	8.40 ± 1.26	9.20 ± 1.93	−1.41	.15
	Transfer	10.00 ± 2.1	11.20 ± 1.68	−1.73	.08
	Total score	65.10 ± 11.68	68.30 ± 10.29	−2.38	.01*

*Note*: Values are expressed as mean ± SD.

Abbreviation: K‐MBI, Korean Modified Barthel Index.

* Significant difference (*p *< .05).

** Significant difference (*p <* .01).

The comparison of the average postintervention changes in the activity of daily living score between the two groups revealed a significant difference in the total score of K‐MBI between the experimental and control groups (*p* < .05). However, there was no significant difference among all subitems (*p* > .05). The AOU and QOM of MAL showed a significant difference between the two groups (*p* < .05) (Table 7).

**TABLE 7:** Comparison of change in activities of daily living scores after the intervention between the groups (*N* = 20)

		Experimental group	Control group		
		Mean ± SD	Mean ± SD	*z*	*p*
K‐MBI	Personal hygiene	0.70 ± 0.67	0.40 ± 0.51	−1.02	.30
	Bathing self	0.80 ± 1.03	0.60 ± 0.96	−0.45	.64
	Feeding	1.40 ± 1.89	0.60 ± 1.26	−1.03	.30
	Toilet	1.20 ± 1.54	0.60 ± 1.26	−0.95	.34
	Stair climbing	0.60 ± 0.96	0.00 ± 1.41	−1.19	.23
	Dressing	0.50 ± 1.08	0.00 ± 1.14	−0.92	.35
	Bowel control	0.80 ± 1.39	0.40 ± 1.57	−0.57	.56
	Bladder control	0.50 ± 1.08	0.00 ± 1.41	−0.92	.35
	Ambulation	0.40 ± 1.26	0.80 ± 1.68	−0.61	.54
	Transfer	2.70 ± 1.88	1.60 ± 2.06	−1.10	.26
	Total score	9.10 ± 6.70	3.20 ± 4.82	−2.62	.00.**
MAL	AOU	6.70 ± 4.19	1.20 ± 0.91	−3.62	.00.**
	QOM	4.80 ± 2.29	1.90 ± 1.37	−2.80	.00.**

*Note*: Values are expressed as mean ± SD.

Abbreviations: AOU, Amount of Use; K‐MBI, Korean Modified Barthel Index; MAL, Motor Activity Log; QOM : Quality of Movement.

* Significant difference (*p *< .05).

.** Significant difference (*p *< .01).

## 4 DISCUSSION

Action observation training is a method of observing a particular action performed by another person and then performing that action during functional training. It is an intervention method that combines the advantageous features of several types of training (Sgandurra et al., 2011). This intervention is used for the rehabilitation process to effectively improve the motor functions of stroke patients and patients with other neurological diseases (Poliakoff, 2013).

The brain activity response to action observation training appears differently depending on the observation perspective (Watanabe et al., 2011). Pelosin et al. (2013) applied brain imaging techniques to investigate the cerebral activity response according to the observation perspective point (Pelosin et al., 2013). Authors found that brain activity responses appearing while observing actions from a first‐person perspective were similar to those observed when moving one's extremities while the brain activity response from observing actions from third‐person perspective was to that when observing other people's movements. Moreover, the model's limbs in the video and the observer's limbs appear to match in anatomical characteristics when action observation is from the first‐person perspective, whereas the model's limbs appear as in looking in a mirror when action observation is from the third‐person perspective. Koski et al. (2003) reported that mirror neurons get more easily activated when mimicking a movement after observing a model in a video that matches the observer's visual perspective in action observation training (Koski et al., 2003).

However, studies that compare different effects from different observation perspectives when applying action observation training to stroke patients are rare. Thus, this study aimed to propose a method of action observation training with increased effectiveness by comparing the effects of action observation training according to the observation perspectives of chronic stroke patients, that is, the first‐person perspective relative to the third‐person perspective. Videos that had different perspectives of observing the action were applied to study subjects; that is, action observation training of the first‐person perspective was applied to the experimental group, while the third‐person perspective was applied to the control group. The results of the assessments are as follows.

This study compared upper extremity functions and the ability to perform activities of daily living before and after the intervention and found significant differences in the ARAT, K‐MBI, and MAL evaluations for both the experimental and control groups (*p* < .05). The ARAT evaluation showed significant differences in all items in the experimental group (*p* < .05) and showed significant differences in all items except pinch in the control group (*p* < .05). Regarding the K‐MBI score, the total score before and after the intervention showed significant differences for both the experimental and control groups (*p* < .05). Regarding the subitems, four subitems, such as personal hygiene, feeding, toilet transfer, and dressing, showed significant differences in the experimental group. Two subitems, such as personal hygiene and dressing, showed significant differences in the control group (*p* < .05). In addition, the AOU and QOM items of MAL showed a significant difference in both the experimental and control groups (*p* < .05).

Stefan et al. (2005) reported that motor learning could be promoted when the action observation method and practical physical exercise were combined (Stefan et al., 2005). According to McGregor and Gribble (2015), action observation training can promote motor learning by activating neuroplasticity in the sensory area and motor area in the cerebrum, and observing an action and then mimicking it is an effective intervention for reconstructing and maintaining a loop circuit that connects the motor cortex, basal ganglia, thalamus, and cerebellum (McGregor & Gribble, 2015). In the aforementioned studies, the upper extremity function and the ability to perform activities of daily living improved in both the experimental and control groups. Moreover, these results are consistent with the results from the following studies. Jo, Bang, Lee, Bang and Son (2011) found that the functional level of the upper extremities and hands was significantly different after the action observation training in chronic stroke patients (Jo, Y.S. Bang, Lee, J.H. Bang & Son, 2011). Bae and Kuk (2012) found significant differences in both upper extremity functions and activities of daily living performance after the application of action observation training among chronic stroke patients (Bae & Kuk, 2012).

In comparing postinterventional changes of scores in upper extremity functions and activity of daily living between the two groups, the experimental group showed more significant changes in the ARAT, K‐MBI, and MAL than the control group (*p* < .05). Specifically, in ARAT, the change was more significant than all sub‐items except for the total score and the pinch (*p* < .05). The change in the total score of K‐MBI was also statistically significant in the experimental group in comparison to the control group (*p* < .05) and for the AOU and QOM items of MAL in the experimental group (*p* < .05). Overall, the group comparison showed more significant improvement in the experimental group than in the control group for most of the upper extremity functions and activities of daily living (*p* < .05). Such findings indicate that first‐person perspective action observation is more effective than third‐person perspective action observation, which is consistent with the results from previous studies.

Jackson et al. (2006) found a higher activity level in the sensorimotor cortex during first‐person perspective observation than third‐person perspective observation (Jackson et al., 2006). Watanabe et al. (2011) reported that first‐person perspective action observation could effectively promote motor learning because it induces more localized and selective cerebral activation than third‐person perspective action observation (Watanabe et al., 2011). Giorgi et al. (2018) compared the therapeutic effects of first‐person perspective action observation training versus third‐person perspective action observation training in patients with Parkinson's disease (Giorgi et al., 2018). The authors reported that first‐person perspective action observation training improved their upper extremity function more effectively than third‐person action observation training. Similarly, in this study, the intervention was more effective in the experimental group that was subject to action observation training from the first‐person perspective than the third‐person perspective because the viewer imitated the movements after observing a model in the video from a visual perspective matching their perspectives.

Fadiga et al. (1995) reported difficulties of action observation training in improving fine movements, such as pinching. Our study did not show a significant postintervention difference in the effect on the pinch item between the experimental group and the control group (Fadiga et al., 1995). However, in the before and after group comparison, the control group with action observation from a third‐person perspective did not show a significant improvement in pinching. In contrast, post‐intervention improvement was present for the pinch item in the experimental group that was subject to first‐person perspective action observation. Such a result is deemed to have resulted from the different visual perspectives of an observer, which is consistent with the findings of Pelosin et al. (2013) that the greater the anatomical matching between the observed action and observer's action, the easier it is to imitate the movement, facilitating motor learning more effectively (Pelosin et al., 2013). As a result, the experimental group showed a more significant post‐intervention change than the control group for most of the upper extremity functions and activities of daily living (*p* < .05). This is in congruence with the study by Giorgi et al. (2018) that reported that first‐person perspective action observation training was more effective in improving upper extremity function than third‐person perspective action observation training for patients with Parkinson's disease (Giorgi et al., 2018).

In our study, the experimental group was subject to first‐person perspective action observation training and tried to imitate the movement after observing the model in the video from the observer's visual perspective. The intervention was more effective than that in the control group, where a third‐person perspective action observation was applied. Therefore, we propose that the first‐person perspective should be used to provide more effective action observation training in the clinical field.

The limitations of this study are as follows. It was conducted with a small number of patients who satisfied the selection criteria in a single hospital in a particular region over a short intervention period. Thus, there are limitations in generalizing the study results to all stroke patients. Future studies should be conducted with a larger sample, and research should be conducted by taking individual characteristics into account. Additional research is necessary on the effects of first‐person perspective action observation training according to the location and type of a lesion or the symptoms. Future studies can be conducted for a more extended intervention period and a larger sample size, providing first‐person perspective action observation training with client‐centered tasks to produce more robust research results.

## 5 CONCLUSION

Subjects of this study were assigned and trained in first‐person action observation training and third‐person action observation training. As a result, better upper extremity function and improvement of daily living performance were reported in subjects who performed first‐person action observation training.

### PEER REVIEW

The peer review history for this article is available at https://publons.com/publon/10.1002/brb3.2565.

## ETHICS STATEMENT

This study was approved by the Institutional Review Board of Cheongju University in accordance with Declaration of Helsinki.

## SOURCE(S) OF FINANCIAL SUPPORT IN THE FORM OF GRANTS (QUOTE THE NUMBER OF THE GRANT) EQUIPMENT, DRUGS, ETC

None.

## STATEMENTS AND DECLARATIONS REGARDING CONFLICTS OF INTEREST SHOULD APPEAR HERE

None.

## ETHICAL APPROVAL

Approved by the Institutional Review Board of Cheongju University (1041107‐202004‐HR‐013‐01).

## Data Availability

The date that support the findings of this study are available on request from the corresponding author.
